# An eHealth Platform for the Support of a Brazilian Regional Network of Mental Health Care (eHealth-Interop): Development of an Interoperability Platform for Mental Care Integration

**DOI:** 10.2196/10129

**Published:** 2018-12-07

**Authors:** Newton Shydeo Brandão Miyoshi, João Mazzoncini De Azevedo-Marques, Domingos Alves, Paulo Mazzoncini De Azevedo-Marques

**Affiliations:** 1 Ribeirão Preto Medical School University of São Paulo Ribeirão Preto Brazil

**Keywords:** eHealth, mental health, health information exchange, health information interoperability, medical record linkage, continuity of patient care

## Abstract

**Background:**

The electronic exchange of health-related data can support different professionals and services to act in a more coordinated and transparent manner and make the management of health service networks more efficient. Although mental health care is one of the areas that can benefit from a secure health information exchange (HIE), as it usually involves long-term and multiprofessional care, there are few published studies on this topic, particularly in low- and middle-income countries.

**Objective:**

The aim of this study was to design, implement, and evaluate an electronic health (eHealth) platform that allows the technical and informational support of a Brazilian regional network of mental health care. This solution was to enable HIE, improve data quality, and identify and monitor patients over time and in different services.

**Methods:**

The proposed platform is based on client-server architecture to be deployed on the Web following a Web services communication model. The interoperability information model was based on international and Brazilian health standards. To test platform usage, we have utilized the case of the mental health care network of the XIII Regional Health Department of the São Paulo state, Brazil. Data were extracted from 5 different sources, involving 26 municipalities, and included national demographic data, data from primary health care, data from requests for psychiatric hospitalizations performed by community services, and data obtained from 2 psychiatric hospitals about hospitalizations. Data quality metrics such as accuracy and completeness were evaluated to test the proposed solution.

**Results:**

The eHealth-Interop integration platform was designed, developed, and tested. It contains a built-in terminology server and a record linkage module to support patients’ identification and deduplication. The proposed interoperability environment was able to integrate information in the mental health care network case with the support of 5 international and national terminologies. In total, 27,353 records containing demographic and clinical data were integrated into eHealth-Interop. Of these records, 34.91% (9548/27,353) were identified as patients who were present in more than 1 data source with different levels of accuracy and completeness. The data quality analysis was performed on 26 demographic attributes for each integrable patient record, totaling 248,248 comparisons. In general, it was possible to achieve an improvement of 18.40% (45,678/248,248) in completeness and 1.10% (2731/248,248) in syntactic accuracy over the test dataset after integration and deduplication.

**Conclusions:**

The proposed platform established an eHealth solution to fill the gap in the availability and quality of information within a network of health services to improve the continuity of care and the health services management. It has been successfully applied in the context of mental health care and is flexible to be tested in other areas of care.

## Introduction

### Background

The care of mental disorders frequently requires a coordination of efforts among different health care professionals, services, and care levels (primary, secondary, and tertiary) [1]. There is also a frequent need to integrate the care of mental disorders with the care of nonpsychiatric health problems (such as cardiovascular disorders, diabetes, maternal health, HIV/AIDS, and cancer) for the same people. This type of comorbidity is common, and there is evidence that an adequate management of behavioral problems can positively impact other health conditions [2,3].

However, all around the world, an adequate continuity of care—defined as the coordination and integration of different health care events to meet patient care needs in a coherent, connected, and consistent manner [4]—is commonly affected by the lack of consistent information exchange between different services in care levels [5].

An extensive research area in computer science that seeks to assist in the problem of fragmentation of health care information systems is interoperability and data integration. The Institute of Electrical and Electronics Engineers Standards Computer Dictionary defines interoperability as “the ability of two or more systems or components to exchange information and to use the information that has been exchanged” [6]. In the health care domain, Healthcare Information and Management Systems Society (HIMSS) defines interoperability as the ability of different information systems and applications to communicate, exchange data, and transparently use such data and associated information [7].

Health information systems that offer support to physicians and other health professionals are often not integrated [8], and the exchange of health information relating to the same patient between different care levels and different health institutions is usually nonexistent or incomplete. This can affect the continuity of care and communication among different care providers.

Coffey et al [9] point out that the fragmentation of services undermines mental health care by creating a barrier between different providers and the patient, making it difficult to provide effective treatment. According to Spiranovic et al [10], poor data quality, inconsistencies and discrepancies in information, and lack of interoperability among health information systems are some of the main difficulties for the secondary use of electronic health record data, including the public health management.

Brazil is a middle-income country with the world’s fifth largest population. Since 1988, a complex national health system (highly decentralized) has been implemented, with responsibilities in the funding and management divided between the 3 federated levels (cities, states, and country). The national health system is also composed of a mix of public and private, and profit and nonprofit services [11]. As in other countries, the prevalence of mental disorders is high, and the comorbidity between mental disorders and nonpsychiatric health problems is common [12]. Furthermore, because the regional integration between services is still limited and extremely challenging, there is an urgent need to develop appropriated and feasible integration strategies and solutions for mental health services [13].

### Objective

Considering the need for a computational environment to facilitate the exchange of health information to improve the continuity of care between care levels, the eHealth-Interop computing platform was designed and deployed. The main objective of the eHealth-Interop platform is to provide a service for the exchange of standardized information on mental health, promoting the improvement of the quality of information and its availability in a transparent way for all the regional services responsible for mental health care.

## Methods

### Context of the Study: The Mental Health Care Network of the XIII Regional Health Department of the Sao Paulo State, Brazil

In Brazil, public mental health care has to be carried out through regional public mental health care networks (MHCNs), whose objectives are to guarantee the continuity and integrality of care through the different services and care levels [14].

The XIII Regional Health Department (RHD) covers over 26 municipalities [15], with approximately 1,400,000 inhabitants [16], where Ribeirão Preto is the main city. The development and current features of its MHCN are described in detail elsewhere [17-19]. Briefly, it comprises (1) primary health care, community-based mental health, and emergency services managed by the 26 municipalities; (2) acute inpatient psychiatric beds, outpatient specialized clinics, and a day hospital located at 2 state psychiatric hospitals and 1 state university general hospital; and (3) long-term residential mental health care facilities managed by municipalities and the state.

The extreme financial and administrative decentralization of public health services in Brazil (with the different health services, within a same health region, managed either by the municipal or by the state or even by the federal sphere) results in enormous heterogeneity of computerized information systems used by different services and professionals. For example, within the XIII Region of Health of the State of São Paulo, each of its 26 municipalities has 1 specific information system. Furthermore, each state-sphere health service has its own information system. The same happens with the private health services hired by the municipal or state public authority. Each one has its own computerized information system that is not shared with other services.

In the last 5 years, the Brazilian Ministry of Health has made available for primary care–level services a computerized information system called e-SUS AB [20] as part of a strategy to improve the interchange and integration of health care information. For the psychiatric care, referral and counter-referral in the region are articulated at the regional level through an information system called SISAM (*Sistema de Informação em Saúde Mental*; in English, mental health information system) [17], which has a module for appointments scheduling in community mental health care units, too.

Although the different services present in the network have mechanisms to integrate care between them, as recommended by Brazilian health legislation (such as the SISAM itself and periodic meetings between services representatives) [17,18], from the informational and computational point of view, support systems of the different services are not integrated. This situation presents an immense difficulty for the health information exchange (HIE) between different services and professionals necessary for the efficient and safe management of the needs and demands of each health system user.

Therefore, information loss, data duplicity, lack of consistency, and rework are common. In this scenario, the work reported here was focused on the integration of 5 information sources from different information systems that support MHCN in the various care levels. The information sources are as follows: SISAM; e-SUS AB; data from 2 state psychiatric hospitals; and access to Web services of the Brazilian Ministry of Health for integration, with patients’ demographic data through the national health card.

### Interoperability Information Model

**Figure 1:**
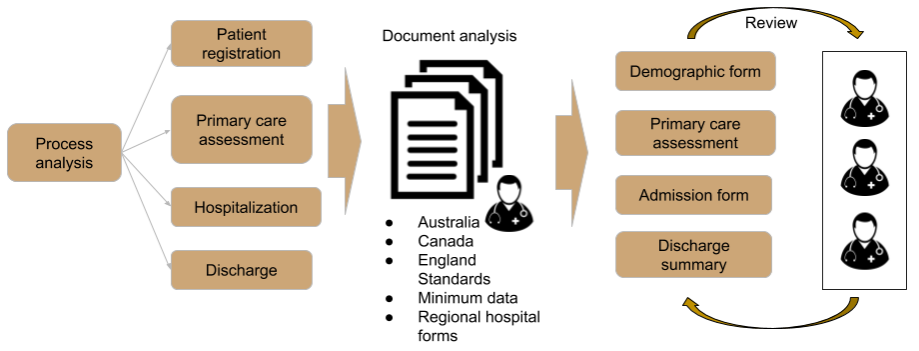
Process for generating the interoperability information model.

In the eHealth-Interop platform, the interoperability data model corresponds to the information set that is exchanged and made available to health professionals at different care levels. To define this set of information, we analyzed the main processes involved in patient care. The identification of the main processes occurred in meetings with the professionals involved in the MHCN using a sociotechnical approach. With this approach, potential users and technical developers act together during the planning, development, and implementation of computational solutions [21,22]. The professionals were (1) clinicians from specialized hospitals, community mental health services, and primary health care; (2) municipal and regional health services managers; and (3) specialized technicians in health informatics. From these meetings, possible care events were identified and prioritized to share information. Moreover, 4 different events were highlighted: (1) patient entry into the network care, (2) assessment in primary care, (3) the admission in psychiatric hospital service, and (4) the discharge of these services. For each process, a set of data elements was defined based on analysis of existing documents. Finally, proposed data elements were reviewed by mental health specialists for information inclusion or exclusion (Figure 1).

The documents analyzed include records used in MHCN logistic instruments, national standards, and international documents. The records used are SISAM demographic form and a regional mental health specialized hospital discharge summary. Furthermore, the minimal health dataset structured by the Brazilian Ministry of Health was analyzed [23], together with the discharge summary proposed by the *Associação Brasileira de Normas Técnicas* (Brazilian Association of Technical Standards technical group on health informatics) [24].

Besides this set of national documents, documents from the United Kingdom, Canada, and Australia were also analyzed. From Australia, the following documents were analyzed: national minimum dataset—admitted patient mental health care [25], national minimum dataset—residential mental health care [26], and national minimum dataset—community mental health care [27]. From the United Kingdom, the mental health and learning disabilities dataset [28] was analyzed, and the document analyzed from Canada was the common dataset—mental health [29]. We also reviewed the information highlighted in a paper describing minimum data to continuity of care [30].

As a result of the described process, a set of information was defined for each of the 4 events: demographic form, with patient demographic and identification information; primary care assessment, with clinical information from primary care; admission form, with information of the hospitalization request and the admission moment; and the discharge summary, with data of the whole hospitalization process. These information sets were reviewed, reformulated, and approved by a group of around 20 professionals, representative of the several health services and municipalities involved in MHCN.

### Interoperability

The defined interoperability data models serve as a conceptual basis for the HIE. The eHealth-Interop platform acts as a central repository of the data exchanged. All communication is performed through Web services available at the eHealth-Interop. Each data source, which is the information system in the different health services, sends and queries data from its patients through Web service clients that communicate with eHealth-Interop.

The process of sending patient data can be compared with the extraction-transformation-load steps in a classical data integration process. First, the data of interest are extracted from the local databases in the format that is specific to each information system. Then, the data are transformed to the interoperability data model previously defined. These transformed data are then uploaded to eHealth-Interop using specific end points provided by the platform Web services application programming interface (API). Thereafter, the data undergo a syntactic and duplicity analysis, aiming to improve the quality of the information. If the patient’s record is already recorded in eHealth-Interop, the new information will be aggregated, making a more complete record.

### Data Quality Analysis

The analysis of the effectiveness of quality improvement, obtained from data integration provided by the proposed interoperability solution, was performed through the measurement of data quality. Data quality metrics used were as follows:

- Completeness represents the degree to which a given collection of data has the data it should represent. Data completeness is defined as the level of missing data for a data element
- Syntactic accuracy represents the degree to which the given data are correct and reliable. Syntactic accuracy measures the degree to which the given data correspond to a possible value in the dataset, for example, age must be between 0 and 120 years
- Duplicity represents the degree to which the given data are unnecessarily duplicated within a dataset of interest, for example, 2 or more records belonging to a same record in the same data source

The data quality metrics of completeness and syntactic accuracy were calculated considering the data sources in 3 different moments during the data interoperability process. In the first moment, the data are available based on as they are originally represented in the different data sources. In a second moment, the data are available considering the normalization process into the data source adapters. During this process, each value of each attribute is transformed (if necessary) to conform to the defined interoperability data model. In a third moment, different records belonging to the same patient, which are originally in different sources, are integrated into a single record, and then the quality metrics are applied over them.

The duplication analysis was performed to verify the use of the record linkage (RL) algorithms and was applied over a set of records in the second stage that did not have deterministic identifiers.

### Health Data Standards

During the design and implementation stage of eHealth-Interop and its components, 2 health information standards served as the basis: the Integrating the Healthcare Enterprise (IHE) and the Health Level-7 Fast Healthcare Interoperability Resources (HL7-FHIR).

IHE is a joint initiative initially formed by the HIMSS and Radiological Society of North America in 1988 [31]. The IHE aims to coordinate the use of health information standards, terminologies, and protocols to enable plug-and-play interoperability between systems from different vendors. The IHE Patient Identifier Cross-Referencing (PIX) profile is an integration profile that defines a methodology for cross-referencing patient identifiers from different sources [32].

FHIR is an HL7 standard that seeks to enable the construction of effective interoperability between applications using Web development standards [33].

The general principles of development enforced by HL7-FHIR, such as RESTful architecture; use of Web development standards such as JavaScript Object Notation (JSON) and open authorization (OAuth); and definition of concise and flexible data models have been adopted for the management of clinical and demographic data in eHealth-Interop. The HL7-FHIR terminology module specification was implemented for the eHealth-Interop terminology server. The IHE PIX profile served as the basis for the overall planning of the patient identification process, although not completely adopted. In this context, eHealth-Interop acts as the patient actor identifier cross-reference manager, and we rely on the patient identity feed, patient identity management, and PIX query transactions to define some of the services responsible for managing demographic data.

## Results

### eHealth-Interop Architecture

The architecture of a computational system seeks to define the general structures such as its main elements, the relationships between these elements, and the properties of the elements and these relationships [34]. According to Soni et al [35], the conceptual architecture of a system defines a high-level structure and is independent of the implementation and technical decisions.

**Figure 2:**
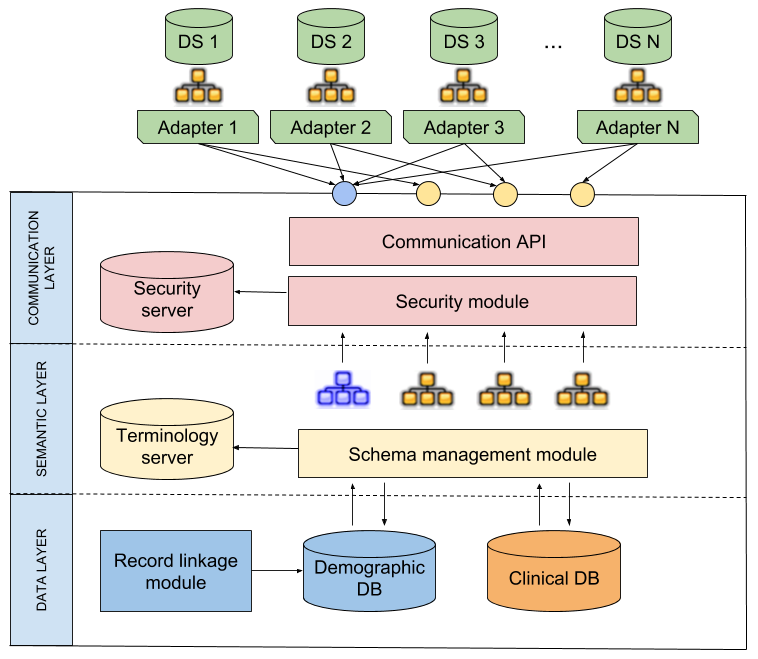
Conceptual architecture of eHealth-Interop. API: application programming interface; DB: database; DS: datasource.

Taking into account that regional care delivery is made up of different actors located at different points in a given region, the Web environment was defined as the basis for the integration platform following a client-server architecture. The proposed conceptual architecture of eHealth-Interop is shown in Figure 2. The architecture follows a model with 3 main layers: data layer, semantic layer, and communication layer. The data layer is where the data to be integrated are represented and stored; semantic layer, in which the mediating schemas, terminologies, ontologies, local codifications, and mappings between them are described; and finally, the communication layer, where security aspects and communication with the data sources are managed.

Each layer has specific components. Functionalities of each component are described below.

In the data layer, data are stored following a dual model, ensuring the differentiation between demographic and clinical information. This distinction seeks to establish a solid foundation for patient identification algorithms and at the same time make the database flexible to be extended to different application contexts in the medical field [36]. Another important component of the data layer is the RL module. This module is responsible for ensuring that there is no duplication of patient registration through the information provided in the demographic database.

The semantic layer is mainly composed of a terminology server responsible for the storage of codifications, terminologies, and ontologies as well as their relationships. It also comprises the clinical and sociodemographic concepts that compose the information models and the schema management module that connects the information model with the terminologies and communicates with databases.

The communication layer is responsible for generating the service API from the information models represented in the semantic layer and for promoting a secure communication by the security module that verifies the authentication and authorization of the person responsible for the process of data sending and retrieval. The exchange of information from the data source to the interoperability platform is done through adapters that are software agents that translate the source schema into the global model provided by the service interface.

### Terminology Server

Medical vocabulary is highly heterogeneous, and several information artifacts are constantly emerging aiming to standardize not only medical concepts but also relationships between them. These artifacts are usually named codifications, terminologies, controlled vocabularies, and ontologies, among others [37]. The terminology server is a software component that provides several services for metadata management related to medical concepts. In HIE context, the terminology server is important to ensure not only the standardization of terms but also the information quality being integrated. The eHealth-Interop terminology server was designed based on the terminology specification of the HL7-FHIR and has part of its design based on the Object Management Group common terminology services 2 [38] specification.

**Figure 3:**
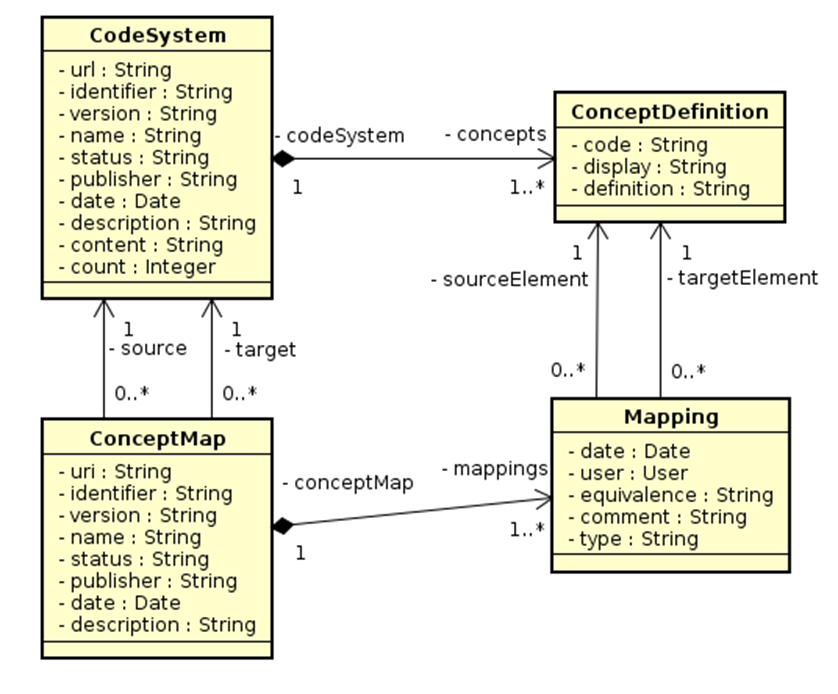
Terminology server components.

The main classes implemented are CodeSystem, ConceptDefinition, ConceptMap, and mapping (Figure 3). CodeSystem represents a particular ontology or terminology; it represents the total set of codes and their meanings. Concepts represent a particular concept or term in an ontology or terminology. ConceptMap represents a mapping between two CodeSytems or part of it. A mapping represents a relationship between two concepts from different CodeSystems.

In the medical context, or specific mental health, CodeSystem represents more complex terminologies such as Systematized Nomenclature of Medicine (SNOMED) [39], classifications such as 10th revision of the International Statistical Classification of Diseases and Related Health Problems (ICD-10), and even local value sets such as to represent sexual orientation. ConceptDefinition are specific concepts, for example, the diagnosis of ICD-10, F03-Dementia. Mapping can represent the mapping between two ontologies such as CID10 and SNOMED, or the mapping between a local encoding (sexual orientation) and SNOMED, for example. ConceptMap are the individual concept-to-concept mapping, for example, dementia (F03) of the ICD10 that can be mapped as equivalent to the concept of dementia disorder (C0497327).

To support the information model defined for continued care in the MHCNs, 5 terminologies have been used:

- ICD-10
- International Classification of Functioning, Disability, and Health
- International Classification of Primary Care
- Table of Procedures, Medications and Orthotics, Prostheses, and Materials of the Unified Health System in Brazil
- Brazilian Classification of Occupations

In addition, local value sets were created to represent different concepts such as sex, marital status, and sexual orientation, among others. These codifications, for the most part, are in agreement with those used in the national information system for primary care or based on information models from a national standard for discharge summary.

### Record Linkage Module

RL can be defined generally as the process of identifying records from different sources that correspond to the same real-world entity [40]. The RL process is also known as entity resolution, deduplication, entity matching, merge or purge problem, data reconciliation, or in a clinical context as patient matching, among others. The RL process is essential to ensure the integrity of information and data quality during HIE and data integration.

The health information system usually implements the RL based on a deterministic method in which certain precise rules are defined to guarantee the uniqueness of a patient’s record. In mental health context and in other areas such as medical emergencies, it is often not possible to obtain a unique identification or reliable information from patients. Therefore, using a deterministic method for patient RL is not always possible. In addition, duplicity may occur from erroneous manual registration by users operating health information systems. These errors and lack of information can be propagated in a scenario of HIE in which several systems are involved.

**Figure 4:**
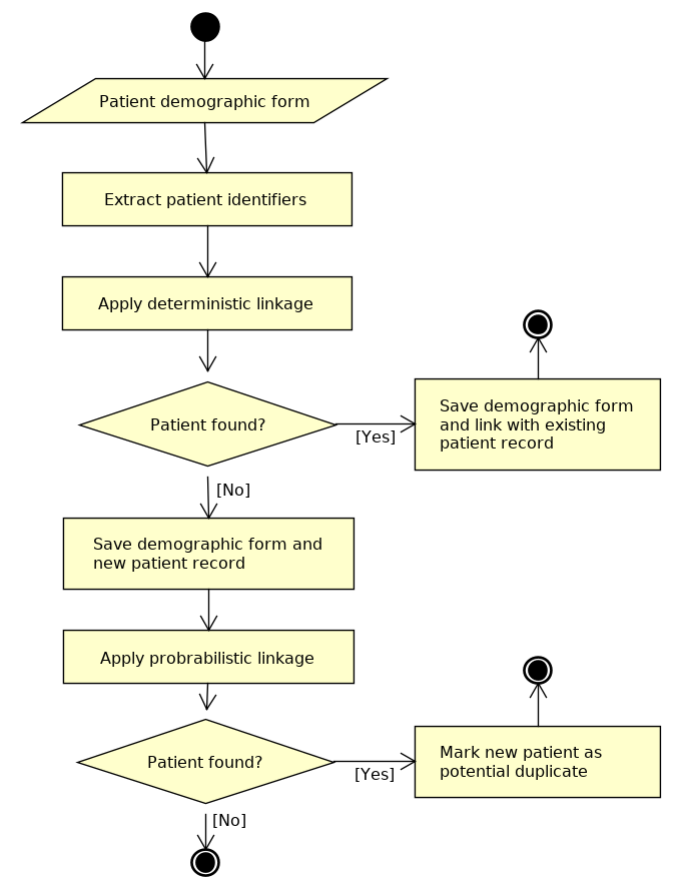
Information flow during patient registry in eHealth-Interop.

To solve this kind of inconsistency, eHealth-Interop comprises an RL module that uses a 2-stage verification to ensure the uniqueness of the patient record. This process is summarized in Figure 4. The first step is to extract patient identifiers, for example, patient’s national identifier or health record number and apply the deterministic linkage using those identifiers. If a patient is not found, probabilistic linkage algorithms are applied using demographic information. When probabilistic linkage algorithms find a possible duplicity of identity, this duplicated record is assigned to a human audit process in which an expert analyzes the information and decides if the duplication really occurred or not.

### Security Server

Information security and privacy are important aspects of any information system. In the medical field, it usually becomes a major barrier for the exchange of health information between different institutions [41]. The concern with information security and privacy was considered in different stages during the eHealth-Interop development, that is, in the conceptual design of the integration platform architecture, for data exchange mechanism adoption, and also for the definition of the user data access hierarchy.

The division of the data layer architecture in 2 different repositories, which store patient demographic information and clinical information separately, allows these repositories to be made available on different physical locations with different access methods, increasing security at system level.

All data access is done through Web services that use the HTTPS protocol, establishing a secure communication channel between the client and the server, where the data are encrypted [42]. Every client application to interoperate with eHealth-Interop platform must be previously registered, as well as the users who will access the data through this application. HTTPS ensures that information exchanged in eHealth-Interop is encrypted during the process of transfer between clients. Data stored in databases, by default, is not encrypted because of performance improvements. Nonetheless, all patient unique identification information is stored separately with more restricted access to ensure the safety of this information.

A hierarchy of user access was also implemented following the model already defined by MHCN professionals. In this model, the professionals of the municipal health services have access to the patient’s data only from their own municipality, allowing these data to be accessed by another service in case of a transfer. The regional hospital services have access to the data about those patients who are referred to them. The access is provided based on the OAuth2 protocol [43]. The data privacy model adopted follows the model already established in MHCN and based on the model established by the Ministry of Health in Brazil for primary care. In this case, all professionals involved in patient care have access to shared data. For example, for a patient who underwent treatment in a mental health clinic and then was admitted to a specialized hospital, practitioners from both facilities have access to the patient’s information. The eHealth-Interop enables the definition of stricter or permissive data access strategies depending in the application context. All actions are stored in a logging system, allowing the establishment of an audit trail.

### Communication Application Programming Interface

**Figure 5:**
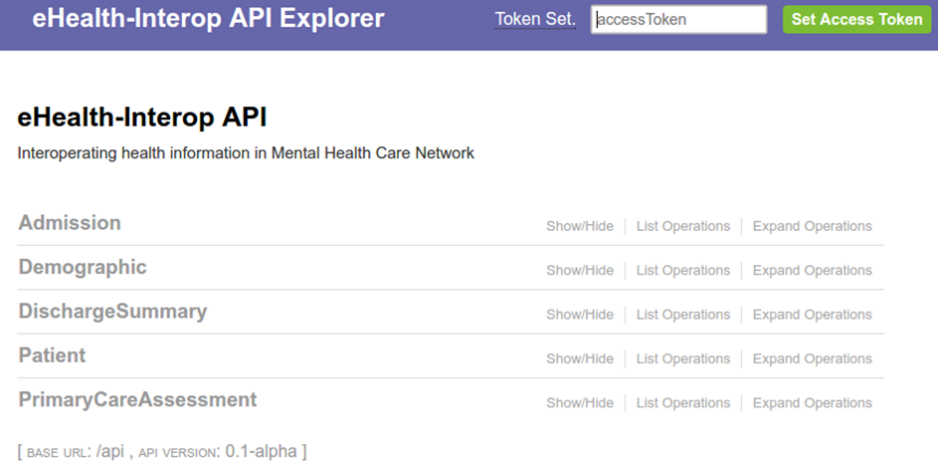
eHealth-Interop Web services API description tool. API: application programming interface.

All communication between the different information sources and the eHealth-Interop platform is done through Web services. The Web services provide a series of functionalities for registration and query of patient demographic data in the platform as well as all the clinical information associated with them. Clinical information is represented on information model defined *a priori* in clinical documents that are exchanged during different moments of the patient care process. A Web tool was also developed to assist the construction of adapters displaying all the services available in eHealth-Interop as well as how to access them (Figure 5).

**Figure 6:**
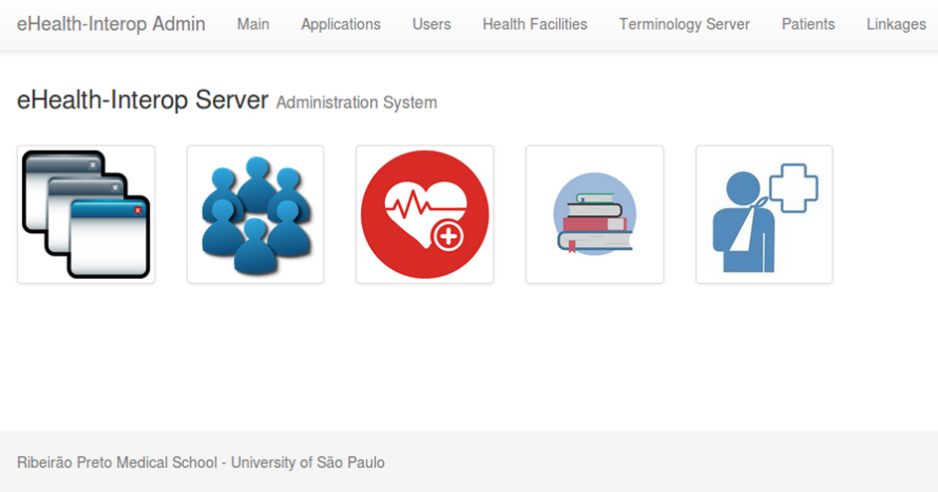
eHealth-Interop administration system.

In addition, a Web administration application was also built (Figure 6). Through this Web administration application, it is possible to manage all users and applications that have access to eHealth-Interop as well as the terminology server and to audit possible duplicate records found by the RL module.

**Figure 7:**
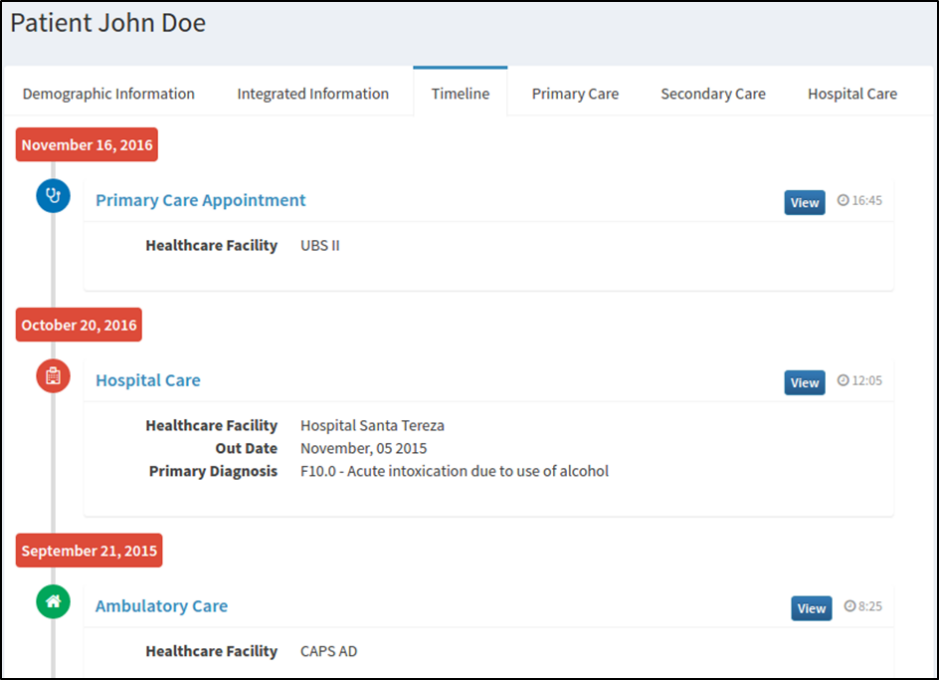
Web application tool to access integrated health care information.

### Integrated Care Web Application

To allow the analysis of the integrated information, a Web application tool was created that acts as client of the eHealth-Interop server. This tool is coupled with SISAM, allowing access to all professionals who work in mental health in the region, based on reuse of predefined and agreed access levels. In this way, access for patient demographic information and clinical information about appointments in the different care levels (primary, secondary, and tertiary) are provided. 

This Web application also provides access to a timeline of patient care events (Figure 7).

### Technical Details

The implementation of this conceptual architecture was made using the LoopBack framework [44] and was deployed using Node.Js programming language. In the data layer, the separation in 2 databases, relational and nonrelational database, was mainly because of 3 reasons. First, all demographic and security information is stored in the relational database using MySQL [45]. This set of information is fixed and is less likely to be altered over time. Second, clinical information grouped in documents that represent health events is more flexible and can be modified according to the clinical context. The data model for representing these documents is based on JSON schema. For this, the nonrelational database MongoDB [46] was chosen, seeking greater flexibility and adaptability of the data model. Finally, this division also allows greater security and anonymity of information, as the database may be available in different locations.

The software API Dedupe [47] was used for the RL module implementation. The Web services application was built using the OpenAPI specification standard [48]. The eHealth-Interop admin Web application was built using the Angular version 2, which is a typescript framework to build Web applications.

The eHealth-Interop performance with the dataset available for testing was satisfactory, with no delay during its use (less than 1 second time of response). It is also important to consider that the set of events that generate access to eHealth-Interop simultaneously is low. Stress testing is needed for scenarios where a much larger volume of data is used concurrently.

### Use Case Example in the Mental Health Care Network

A total of 27,353 patient health records were obtained from the 5 data sources used in this work for a period from 2011 to 2016. Out of these, 9547 (34.90%, 9547/27,353) records have been integrated in eHealth-Interop platform. In these records, the same patient is present in at least two different sources of information with the same identification data. The total number of unique patient records after integration was 4252, which means there were approximately 2.25 records per patient in the original dataset. The number of data sources having the same patient’s record are presented in Table 1.

Completeness and syntactic accuracy analyses were performed over patients’ demographic data. In total, 26 different attributes have been analyzed. For each attribute of each record, a value of 1 or 0 of completeness was assigned, based on the presence or absence of any data. Thereafter, the syntactic accuracy analyses were performed for those attributes with completeness value equals to 1. The syntactic accuracy analysis was done at attribute level for each patient record. Rules of syntactic analysis were defined for each attribute. Some of these rules are demonstrated in Table 2. Completeness and syntactic accuracy analyzes were performed field-by-field of the 9548 integrable records, totaling to 248,248 (9548 × 26) analyzed fields.

Then, each attribute value was tested according to the defined rule of the respective attribute. For example, if the patient has the birth date as February 2, 1970, then the rule is applied to verify if this value is a valid birth date. If the value passes in the test, then it has a value of syntactic accuracy equal to 1, otherwise the value will be zero. Table 3 shows such analysis for all demographic dataset before and after data integration in eHealth-Interop.

Results presented in Table 3 refer to the total set of integrated demographic information. Tests were improved by splitting demographic information into subgroups to better understand how eHealth-Interop acted over data quality. Each subgroup consists of a set of related information. Four subgroups were defined as follows: general, identification, contact, and address. The *general* subgroup consists of general demographic information such as name, mother’s name, father’s name, gender, and date of birth. The *identification* subgroup is composed of key attributes for patient unique identification, including identifiers used at national and local health level. The *contact* subgroup includes residential telephone, cell phone number, and email. The *address* subgroup includes the residence address information of the patient such as city name, street name, and house number. Tables 4 and 5 show the completeness and accuracy analysis, respectively, for each subgroup before and after the data integration through eHealth-Interop.

In case of inconsistent data (2 different birthdates) when performing the entity integration, the value that is syntactically correct is adopted. If both values pass the test, the one from the record with highest value of completeness is adopted. Nevertheless, all the original data from both integrated records are stored before the integration. It is possible to change the canonical record later.

For duplicity analysis, a cured dataset was organized with records that did not have unique identifiers. Then, a semiautomatic strategy was conducted: initially the RL module was used to perform a preselection of possible duplications and then, in the next step, each duplication result was manually verified for the determination of a true positive set of duplicates.

**Table 1:** Distribution of the number of integrable patient records per data source.

Number of data sources having the same patient’s record	Number of unique patient’s records (n=4252), n (%)	Total of integrated records (n=9548), n (%)
2	3247 (76.36)	6494 (68.02)
3	968 (22.77)	2904 (30.41)
4	35 (0.82)	140 (1.47)
5	2 (0.05)	10 (0.10)

**Table 2:** Example of syntactic rules used in the syntactic accuracy analysis.

Attributes	Syntactic rule
Birthdate	A valid date, considering that maximum age is 120 years
Municipality of residence and birth	A name of a registered Brazilian municipality
National health card and national identification number	Predefined numeric rule to validate the specific field
Cell phone and residential telephone number	A numeric value of possible length of a valid Brazilian phone number

**Table 3:** Completeness and accuracy analysis in the whole dataset of integrated records (n=248,248).

Measures	Completeness			Accuracy		
	Nonintegrated, n (%)	Integrated, n (%)	Difference	Nonintegrated, n (%)	Integrated, n (%)	Difference
Minimum	0 (0.00)	0 (0.00)	—^a^	137,580 (55.42)	170,472 (68.67)	+13.25
Maximum	248,248 (100.00)	248,248 (100.00)	—	248,248 (100.00)	248,248 (100.00)	—
Mean	140,186 (56.47)	185,863 (74.87)	+18.40	237,549 (95.69)	240,230 (96.77)	+1.08
SD	83,957 (33.82)	87,234 (35.14)	+1.32	25,023 (10.08)	179,981 (7.25)	−2.83

^a^A dash indicates that no difference was observed.

**Table 4:** Completeness analysis of demographic information divided in subgroups (n=248,248).

Completeness	General integration			Identification integration				Contact integration				Address integration		
	Before, n (%)	After, n (%)	Diff^a^	Before, n (%)	After, n (%)	Diff	Before, n (%)		After, n (%)	Diff	Before, n (%)		After, n (%)	Diff
Minimum	0 (0.00)	3103 (1.25)	+1.25	0 (0.00)	159,102 (64.09)	+64.09	0 (0.00)		8143 (3.28)	+3.28	14,001 (5.64)		48,011 (19.34)	+13.70
Maximum	248,248 (100.00)	248,248 (100.00)	+0.00	234,768 (94.57)	244,251 (98.39)	+3.82	180,973 (72.90)		211,458 (85.18)	+12.28	200,907 (80.93)		246,907 (99.46)	+18.53
Mean	155,329 (62.57)	188,718 (76.02)	+13.45	141,774 (57.11)	210,961 (84.98)	+27.87	108,981 (43.90)		132,813 (53.50)	+9.60	148,924 (59.99)		216,323 (87.14)	+27.15
SD	87,383 (35.20)	87,905 (35.41)	+0.21	99,895 (40.24)	36,368 (14.65)	−25.59	95,973 (38.66)		109,179 (43.98)	+5.32	65,637 (26.44)		74,276 (29.92)	+3.48

^a^Diff: difference.

**Table 5:** Accuracy analysis of demographic information (n=248,248).

Accuracy	General integration			Identification integration			Contact integration			Address integration		
	Before, n (%)	After, n (%)	Diff^a^	Before, n (%)	After, n (%)	Diff	Before, n (%)	After, n (%)	Diff	Before, n (%)	After, n (%)	Diff
Minimum	137,579 (55.42)	170,472 (68.67)	+13.25	237,449 (95.65)	244,499 (98.49)	+2.84	247,801 (99.82)	247,702 (99.78)	−0.04	206,741 (83.28)	215,032 (86.62)	+3.34
Maximum	248,248 (100.00)	248,248 (100.00)	+0.00	248,248 (100.00)	248,248 (100.00)	+0.00	248,000 (99.90)	248,248 (100)	+0.10	248,248 (100.00)	248,248 (100.00)	+0.00
Mean	232,931 (93.83)	236,357 (95.21)	+1.38	243,184 (97.96)	246,510 (99.30)	+1.34	247,900 (99.86)	248,000 (99.90)	+0.04	237,201 (95.55)	239,386 (96.43)	+0.88
SD	34,382 (13.85)	24,527 (9.88)	−3.97	5437 (2.19)	2011 (0.81)	−1.38	149 (0.06)	273 (0.11)	+0.05	17,377 (7.00)	14,374 (5.79)	−1.21

^a^Diff: difference.

The RL algorithm has been configured to take into account the patient’s name and mother’s name because they have 100% of completeness after data integration. The adopted threshold was 0.75 (referring to the probability of finding a duplicity), seeking for a high sensitivity for duplications detection. From a total of 6427 records tested, 1066 duplications were automatically preidentified, and from those, 226 were manually confirmed. The duplicity analysis served as proof of concept of the RL module with the pre-established settings.

## Discussion

### Principal Findings

Coordination and continuity of care at different care levels have been a challenge in many health systems. Major informational barriers for continuity of care are the lack of support information mechanisms to ensure information exchange among health providers. Health information systems are important tools to overcome these barriers, but fragmentation and heterogeneity, particularly prominent in the Brazilian national health system, do not allow to achieve the greatest advantages from their use.

To solve this kind of limitation, a computational interoperable health care platform was designed and deployed to establish an HIE environment. In eHealth-Interop, sociodemographic and clinical information are split into 2 different repositories, aiming to provide data security and extensibility.

In the definition of the interoperability information model, in a joint decision with health professionals, it was decided to consider as much as possible of the defined national standards, while taking into account the other documents already used locally by the services and some international documents as well. Some concepts were out of context with respect to mental health, such as surgical procedures performed and allergies. Despite this, the strategy of keeping this information as optional in the data model was adopted. The standardization and use of international documents allows a comparison with other services and contexts.

Although there are several initiatives for HIE, few of these works are applied in the context of mental health. In this context, some characteristics are quite relevant from the point of view of information sharing, such as a greater number of patient passages by different health services, often generating more readmissions and patient’s demographic information not always reliable, generating patients without unique identifiers. The eHealth-Interop acts as integration middleware, flexible to allow the sharing of information, even in situations of low reliability but having mechanisms to improve quality and ensure entity integration such as the RL module and the terminologies service.

Another important result of this work was the measurement of data quality metrics to gauge the impact of the eHealth-Interop platform in informational continuity of care, as impact of HIE are not yet adequately studied [49,50].

### Limitations

In the mental health use case described, the eHealth-Interop supported HIE between primary health care and mental hospital care, but it was not possible to conduct tests with specialized ambulatory care data. However, the platform is able to automatically generate the Web services API and the database schema to store this information if necessary.

Although the HL7-FHIR standard and the IHE PIX profile have been used as the basis for the definition of eHealth-Interop architecture and the interoperability model, they were not fully adopted. It was not possible to fully adopt the standards, mainly because it would be highly costly to enforce the adaptation by the stakeholders involved. It was then opted for as an intermediate strategy, where the basic principles were adopted but with a more flexible and simplified approach, allowing greater adaptation to the client systems that are part of the MHCNs. A future step will be the conformation of the API involving clinical and demographic data management for the HL7-FHIR standard and not just the terminology service.

Another limitation of this project is that in the mental health use case, there is no mapping between the concepts of the adopted terminologies. The eHealth-Interop supports interterminology mapping; however, there was no need to translate concept from 1 terminology to another for the initial application.

### Related Work

Several reviews analyzing the usage, barriers, facilitators, impact, and cost of HIE have been conducted [41,49-51]. Low data quality is one of the challenges described and a possible cause is poor patient matching process [41]. This issue becomes critical depending on the context, as in the case of homeless patients [52].

In the technical domain, there are several papers proposing the implementation of platforms for the exchange of health information [53-55]. Yuksel et al [53] developed the SALUS platform (Scalable, standard-based Interoperability Framework for Sustainable Proactive Post Market Safety Studies), an ontology-based interoperability framework designed to conduct observational studies from data extracted from different data sources. Moraes et al [54] proposed a methodology for the exchange of information through multi-agent systems based on OpenEHR used for cardiac surgery planning. Rac-Albu et al [55] proposed a method, based on HL7 v2, for exchanging medical documents seeking the interoperability of health data in Romania.

There are few HIE studies in the context of mental health. Cifuentes et al [56] analyzed strategies for care integration between mental health and primary care level. One barrier to achieve this integration is the lack of interoperability between information systems. Shank et al [57] evaluated, through a statewide survey, the behavioral health providers’ beliefs about HIE. The authors concluded that most providers support the use of HIE, although they also worry about the safety and cost of deploying these solutions.

Although there are several studies and proposals for establishing an HIE environment, this remains an open problem, and its use is still limited [58]. In this paper, we described the whole process of development and use of an HIE tool in a challenging medical context, that is, in the case of mental health. In conjunction with key stakeholders, relevant processes were mapped, and interoperability data models were constructed. We described a multilayer conceptual architecture that supports data exchange. This proposal covers two important aspects: the problem of dealing with different health terminologies and the use of a detailed patient identifier process that encompasses patients without unique identifiers. We tested the solution in a real-world environment.

### Conclusions and Future Directions

The eHealth-Interop is a computing platform designed for health information system interoperability. This proposal was successfully implemented and tested in the context of mental health care. This platform has been built to be (1) flexible, so it can be applied in other scenarios and clinical domains and (2) robust, so records of patients with little information can be integrated and completed.

As future work, we intend to support the creation of alerts and automatic warnings based on specific process, for example, when a patient is hospitalized, a warning is generated for a certain health service warning about the event. We also intend to enable semantic querying using a SPARQL end point and implement algorithms to help the process of semantic markup, as well as add similarity functions to the terminology server, allowing a broad approach in concept searching.

## Abbreviations

API: application programming interface
FHIR: Fast Healthcare Information Resource
HIE: health information exchange
HIMSS: Healthcare Information and Management Systems Society
HL7: Health Level-7
ICD-10: International Statistical Classification of Diseases and Related Health Problems
IHE: Integrating the Healthcare Enterprise
MHCN: mental health care network
PIX: Patient Identifier Cross-Referencing
RHD: Regional Health Department
RL: record linkage
SISAM: Sistema de Informação em Saúde Mental
SNOMED: Systematized Nomenclature of Medicine
